# Emerging Hypersexuality in a Patient With Progressive Supranuclear Palsy

**DOI:** 10.1192/bjo.2025.10745

**Published:** 2025-06-20

**Authors:** Pei Ling Lim, Boon Ceng Chai

**Affiliations:** National University Hospital, Singapore, Singapore

## Abstract

**Aims:** Progressive supranuclear palsy (PSP) typically presents with a constellation of motor symptoms, most commonly with frequent falls and gait disturbances. As the disease progresses, cognitive dysfunction and behavioural abnormalities may develop, however hypersexuality in the absence of the usage of dopaminergic agonists is rarely described. We report a case of a 70-year-old male referred to Consultation Liaison Psychiatry for inappropriate sexual behaviour on a background of Progressive Supranuclear Palsy with Predominant Cerebellar Ataxia.

**Methods:** He was admitted to hospital due to inappropriate sexual behaviours resulting in significant caregiver distress. There were increased sexual demands over the past year with other frontal lobe symptoms of hyperorality, apathy, distractibility and motor perseveration. His clinical history, previous investigations and treatments received were reviewed. He was subsequently diagnosed with Major Neurocognitive Disorder due to multiple aetiologies (PSP, Alzheimer’s disease and Frontotemporal lobar degeneration). He was started on trazodone and memantine with improvement and subsequently discharged home.

**Results:** PSP is known to cause frontal lobe deficits affecting executive function, with apathy, impulsivity and disinhibition, but rarely hypersexuality. Hypersexuality is more commonly associated with use of dopaminergic agonists that may be given to address motor symptoms in PSP. It is known that the use of dopaminergic agonists is associated with impulse control disorders such as pathological gambling, hypersexuality and compulsive eating. Further research into how progressive neurodegeneration from PSP affects brain function may shed more light on the emergence of behavioural changes such as hypersexuality.

In the management of hypersexuality, other contributing factors such as boredom, feelings of insecurity and lack of a sexual partner may need to be considered. Non-pharmacological options include behavioural interventions and education of caregivers. Medications may have potential side effects which need to be considered during prescribing. Serotonergic medications such as Selective Serotonin Reuptake Inhibitors are often used due to lower risks and have shown some benefit in reducing problematic behaviour. Other options include antipsychotics, cholinesterase inhibitors and hormonal treatments. It is also important to consider the wellbeing of family and staff looking after the patient as they may be victims of the patient’s sexual behaviour, and provide the necessary support.

**Conclusion:** Hypersexuality is rare in PSP and a thorough review of all possible causes is required. Management may involve both behavioural interventions and pharmacological treatment to aim to reduce inappropriate behaviours.

